# Correlation between end-tidal and arterial carbon dioxide partial pressure in patients undergoing craniotomy

**Published:** 2012-11

**Authors:** Naser Hemmati, Abdol Hamid Zokaei, Ali Karbasforooshan

**Affiliations:** ^*a*^Department of Anesthesiology and Intensive Care, Imam-Ali hospital, Kermanshah University of Medical Sciences and Kermanshah Health Services, Kermanshah, Iran.

## Abstract

**Background:**

Both end-tidal carbon dioxide pressure (ETCO2) is used routinely as an indicator of arterial partial pressure of carbon dioxide (PaCO2) and thus adequacy of ventilation. Accurate determination of the PaCO2 level in neuroanesthesia is quite important because of its effect on cerebral blood flow and also hyperventilation is often used to reduce intracranial pressure in neurosurgical patients. This study was aimed to evaluate the relationship between ETCO2 and arterial PaCO2 in neurosurgical patients undergoing craniotomy to assess the predictive value of ETCO2 as an indicator of PaCO2 level.

**Methods:**

Forty-five consecutive adult patients with inclusion criteria, scheduled to undergo elective craniotomy surgery were enrolled in this prospective study. Measurements of PaCO2 and ETCO2 were performed at three different intervals: Time 1: 10 min after induction of general anesthesia; time 2: after cranium opening prior to dural incision; and at time 3: start of dural closure. All patients received the same anesthetic agent (propofol, sufentanil, atracurium, oxygen). Data were initially analyzed using Pearson’s Correlation to assess the relationship between PaCO2 and ETCO2 at different stages of the operation. A p-value (P) of less than 0.05 was considered significant. The agreement between the measures of CO2 was assessed using Bland-Altman method, where mean difference and average between PaCO2 and ETCO2 were calculated. The 95% confidence intervals for the lower and upper limits of agreement were presented.

**Results:**

A total of 44 patients, aged 18 to 65 years, ASA grades 1 and 2 were participated in the study. Mean difference, standard deviation and correlation coefficient of the parameters were calculated for three time periods. The values for PaCO2, ETCO2, (PaCO2- ETCO2), and correlation coefficient for 10 min after anesthetic induction, prior to dural incision, and start of dural closure were 35.4 ± 3.2, 32.1 ± 3.2, 3.8 ± 2.1, and 0.565, 36.2 ± 3.1, 32.6 ± 3.2,4.8 ± 3.1, and 0.574, and 36.7 ± 2.4, 33 ± 3.2,3.8 ± 2.3, and 0.627, respectively (p less than 0.01 for all analyses). The greatest mean difference occurred just prior to dural incision. The lowest mean difference was observed at 10 min post-anesthetic induction.

**Conclusions:**

To the present study was aimed to correlate between End-tidal and arterial carbon dioxide partial pressure in neurosurgical patients undergoing craniotomy. Findings of this study showed that ETCO2 consistently underestimates the value of PaCO2 during craniotomy indicating that ETCO2 value can be used instead of PaCO2.

**Keywords:**

End-tidal carbon dioxide pressure, Arterial partial pressure of carbon dioxide, Craniotomy


					Volume 4, Suppl. 1 Nov 2012
Publisher: Kermanshah University of Medical Sciences
URL: http://www.jivresearch.org
Copyright:
Congress of Iranian Neurosurgeons, 14-16 November 2012, Kermanshah, Iran
Paper No. 32
Correlation between end-tidal and arterial carbon dioxide
partial pressure in patients undergoing craniotomy
Naser Hemmati a,*
, Abdol Hamid Zokaei a
, Ali Karbasforooshan a
a
Department of Anesthesiology and Intensive Care, Imam-Ali hospital, Kermanshah University of Medical Sciences and
Kermanshah Health Services, Kermanshah, Iran.
Abstract:
Background: Both end-tidal carbon dioxide pressure (ETCO2) is used routinely as an indicator of arterial partial
pressure of carbon dioxide (PaCO2) and thus adequacy of ventilation. Accurate determination of the PaCO2 level in
neuroanesthesia is quite important because of its effect on cerebral blood flow and also hyperventilation is often
used to reduce intracranial pressure in neurosurgical patients. This study was aimed to evaluate the relationship
between ETCO2 and arterial PaCO2 in neurosurgical patients undergoing craniotomy to assess the predictive value
of ETCO2 as an indicator of PaCO2 level.
Methods: Forty-five consecutive adult patients with inclusion criteria, scheduled to undergo elective craniotomy
surgery were enrolled in this prospective study. Measurements of PaCO2 and ETCO2 were performed at three
different intervals: Time 1: 10 min after induction of general anesthesia; time 2: after cranium opening prior to dural
incision; and at time 3: start of dural closure. All patients received the same anesthetic agent (propofol, sufentanil,
atracurium, oxygen). Data were initially analyzed using Pearson's Correlation to assess the relationship between
PaCO2 and ETCO2 at different stages of the operation. A p-value (P) of less than 0.05 was considered significant.
The agreement between the measures of CO2 was assessed using Bland-Altman method, where mean difference and
average between PaCO2 and ETCO2 were calculated. The 95% confidence intervals for the lower and upper limits
of agreement were presented. .
Results: A total of 44 patients, aged 18 to 65 years, ASA grades 1 and 2 were participated in the study. Mean
difference, standard deviation and correlation coefficient of the parameters were calculated for three time periods.
The values for PaCO2, ETCO2, (PaCO2- ETCO2), and correlation coefficient for 10 min after anesthetic induction,
prior to dural incision, and start of dural closure were 35.4  3.2, 32.1  3.2, 3.8  2.1, and 0.565, 36.2  3.1,
32.6  3.2,4.8  3.1, and 0.574, and 36.7  2.4, 33  3.2,3.8  2.3, and 0.627, respectively (p less than 0.01
for all analyses). The greatest mean difference occurred just prior to dural incision. The lowest mean difference was
observed at 10 min post-anesthetic induction.
Conclusions: To the present study was aimed to correlate between End-tidal and arterial carbon dioxide partial
pressure in neurosurgical patients undergoing craniotomy. Findings of this study showed that ETCO2 consistently
underestimates the value of PaCO2 during craniotomy indicating that ETCO2 value can be used instead of PaCO2.
Keywords:
End-tidal carbon dioxide pressure, Arterial partial pressure of carbon dioxide, Craniotomy
* Corresponding Author at:
Naser Hemati : Department of Anesthesiology and Intensive Care, Imam-Ali hospital, Kermanshah University of Medical Sciences and Kermanshah
Health Services, Kermanshah, Iran. Tel: 09121014521, Email: drhemati_37@yahoo.com, (Hemati N.).

				

